# Hemorrhagic Cardiac Tamponade in an End-Stage Renal Disease Patient on Apixaban

**DOI:** 10.7759/cureus.119353

**Published:** 2026-11-09

**Authors:** Anosh Khan, David A Keyes, Sachin Kumar, Linda M Cox

**Affiliations:** 1 Department of Emergency Medicine, Trinity Health, Livonia, USA; 2 Department of Nursing, Trinity Health, Livonia, USA

**Keywords:** apixaban, atrial fibrillation (af), cardiac tamponade case report, end-stage renal disease (esrd), hemodialysis related, hemopericardium, obstructive shock, pericardial effusion (pe), point-of-care-ultrasound

## Abstract

Direct oral anticoagulants are used for stroke prevention in selected patients with atrial fibrillation who receive hemodialysis. Randomized trial data remain limited in this population, and the anticoagulant effect of a given dose may vary considerably between patients. We report a 56-year-old man receiving hemodialysis three times weekly who was taking apixaban 5 mg twice daily and developed persistent hypotension and abdominal pain during dialysis. The early findings suggested dialysis-associated hypotension or sepsis. Emergency department point-of-care cardiac ultrasound showed a large circumferential pericardial effusion, leading to urgent cardiology evaluation for obstructive shock. Computed tomography obtained during the same evaluation described the effusion as large and possibly hemorrhagic. Pericardiocentesis was unsuccessful because most of the fluid was posterior. Shortly after transfer to the intensive care unit, the patient developed pulseless electrical activity arrest and underwent an emergent subxiphoid pericardial window. Approximately 450 mL of bloody fluid was drained, followed by hemodynamic improvement. Cytology showed no malignant cells, and fluid cultures were negative. This case also highlights the uncertainty surrounding the use of full-dose apixaban in patients receiving hemodialysis, particularly after a major bleeding event.

## Introduction

Atrial fibrillation is common in patients with advanced chronic kidney disease and end-stage kidney disease, but anticoagulation is difficult because both stroke and major bleeding risks are high [1]. Apixaban is often chosen because less of the drug is cleared renally than with several other direct oral anticoagulants. Patients receiving dialysis, however, were excluded from the pivotal trials, so dosing in this population largely rests on pharmacokinetic studies, observational cohorts, and small randomized trials [1-9]. Hemorrhagic pericardial effusion causing cardiac tamponade is a rare but life-threatening bleeding event reported with direct oral anticoagulants [10,11]. When hypotension begins during dialysis, clinicians may reasonably first consider volume shifts, infection, arrhythmia, myocardial ischemia, or medication effects. The clinical course described here shows how nonspecific symptoms can delay recognition of tamponade and how bedside ultrasound can redirect the evaluation.

This case was previously presented at the third Annual Michigan State University College of Osteopathic Medicine Research Day on May 13, 2025. It was also presented as a poster at the CHEST 2025 Annual Meeting of the American College of Chest Physicians on October 22, 2025 [12].

## Case presentation

Patient information and initial presentation

A 56-year-old man with end-stage kidney disease on hemodialysis three times weekly presented from an outpatient dialysis center. Other comorbidities included chronic nonvalvular atrial fibrillation, heart failure with preserved ejection fraction, type 2 diabetes mellitus, hypertension, hyperlipidemia, chronic obstructive pulmonary disease requiring home oxygen, and recent hypotension treated with midodrine. Approximately 70 min into dialysis, his blood pressure decreased to 60/40 mmHg. Dialysis was stopped, emergency medical services were activated, and he received ondansetron and a 250 mL normal saline bolus before arrival.

On arrival to the emergency department, he was alert and oriented. He reported generalized abdominal pain, greatest in the left lower quadrant, but denied chest pain, dyspnea, dizziness, syncope, or pressure-like symptoms. Initial vital signs included a temperature of 36.1°C, blood pressure of 87/52 mmHg, heart rate of 77 beats/min, respiratory rate of 20 breaths/min, and oxygen saturation of 96% on 2 L/min via nasal cannula. He appeared tired and had abdominal tenderness without rebound or guarding. The initial ED examination documented no jugular venous distention, whereas cardiology subsequently recorded a jugular venous pressure of 12-14 cm.

The patient had been prescribed apixaban 5 mg twice daily for atrial fibrillation and had taken a dose on the morning of presentation. His medication list did not include aspirin, any other antiplatelet agent, or a nonsteroidal anti-inflammatory drug. He also took midodrine 10 mg as needed when his systolic blood pressure fell below 100 mmHg during dialysis.

Initial evaluation

The following investigations and initial treatment were performed in the emergency department. Initial laboratory studies showed leukocytosis with a white blood cell count of 16.4 K/µL (range: 4.0-11.0 K/µL), anemia with a hemoglobin level of 10.3 g/dL (range: 13.5-17.5 g/dL), and a lactate level that increased from 4.2 to 4.3 mmol/L (range: 0.5-2.0 mmol/L). Venous blood gas analysis showed mixed metabolic and respiratory acidosis, with a pH of 7.24 (range: 7.31-7.41), pCO_2_ of 61 mmHg (range: 41-51 mmHg), and bicarbonate of 22 mEq/L (range: 22-29 mEq/L). Aspartate aminotransferase was 1,167 U/L (range: 13-39 U/L), and alanine aminotransferase was 813 U/L (range: 7-52 U/L), 30 and 16 times the upper limit of normal, respectively, consistent with ischemic hepatitis in the setting of shock. The international normalized ratio was 3.1 (range: 0.8-1.2), while the activated partial thromboplastin time was normal (22-36 s). A heparin-calibrated anti-factor Xa level was 0.64 IU/mL (therapeutic reference range: 0.6-1.10 IU/mL), consistent with anti-factor Xa activity; an apixaban-specific assay was not performed. In a patient taking apixaban and developing hepatic injury, these tests did not quantify the anticoagulant effect. High-sensitivity troponin was normal (<0.05 ng/mL), B-type natriuretic peptide was mildly elevated (0-100 pg/mL), and testing for SARS-CoV-2, influenza, and respiratory syncytial virus was negative. The admission ECG showed atrial fibrillation at 78 beats/min with right bundle branch block, without acute ST-segment elevation or electrical alternans (Figure 1).

**Figure 1:**
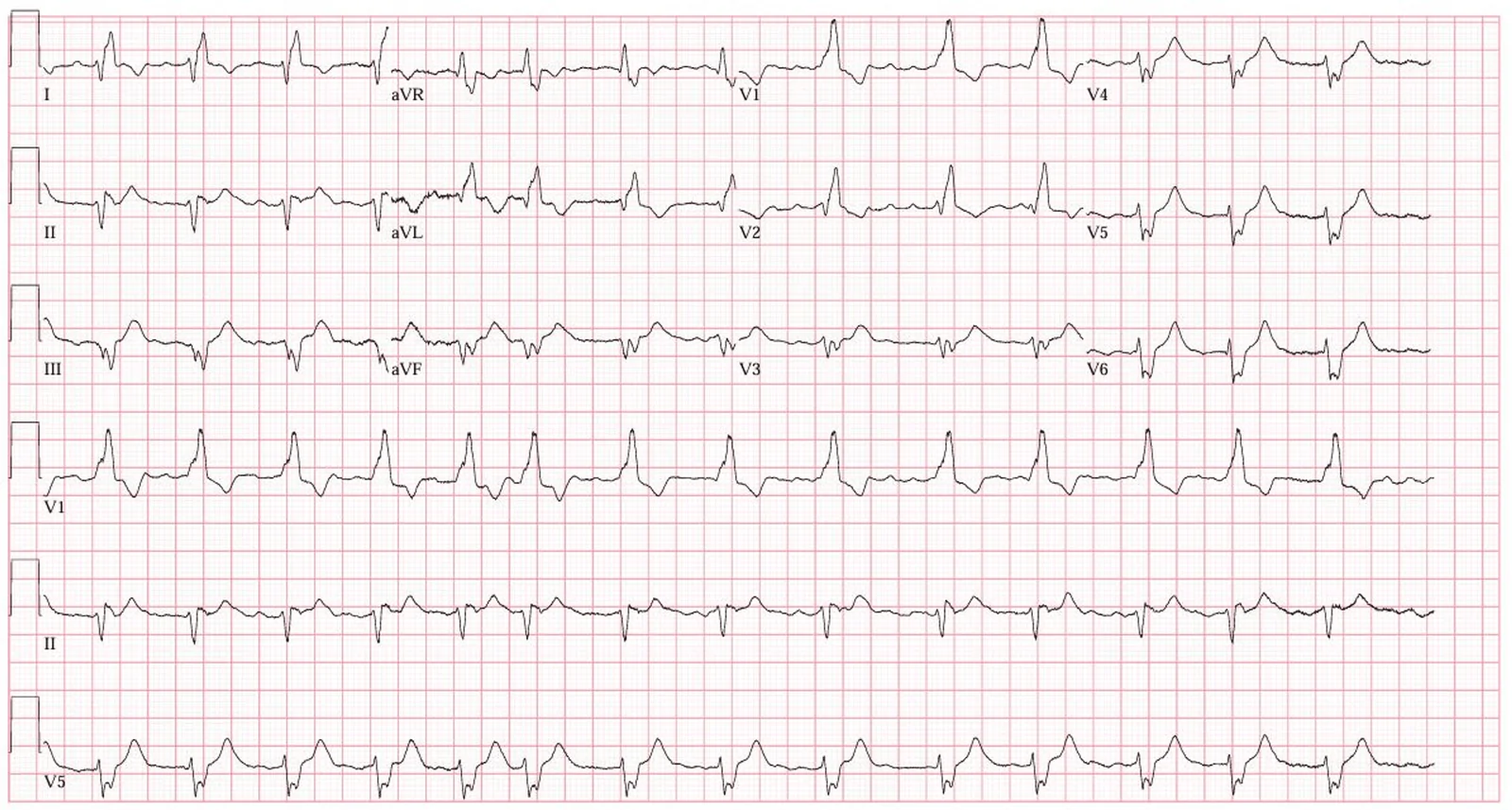
Admission 12-lead electrocardiogram shows atrial fibrillation with right bundle branch block. No clear electrical alternans is evident.

Portable AP chest radiography showed an enlarged cardiac silhouette and mild vascular congestion without overt pulmonary edema, pneumothorax, or a large pleural effusion (Figure 2). Sepsis was initially considered, and empiric broad-spectrum antibiotics were administered while the evaluation continued. Leukocytosis and elevated lactate were nonspecific and did not distinguish the cause of shock. Fluid administration was cautious because of end-stage kidney disease and concern for volume overload.

**Figure 2:**
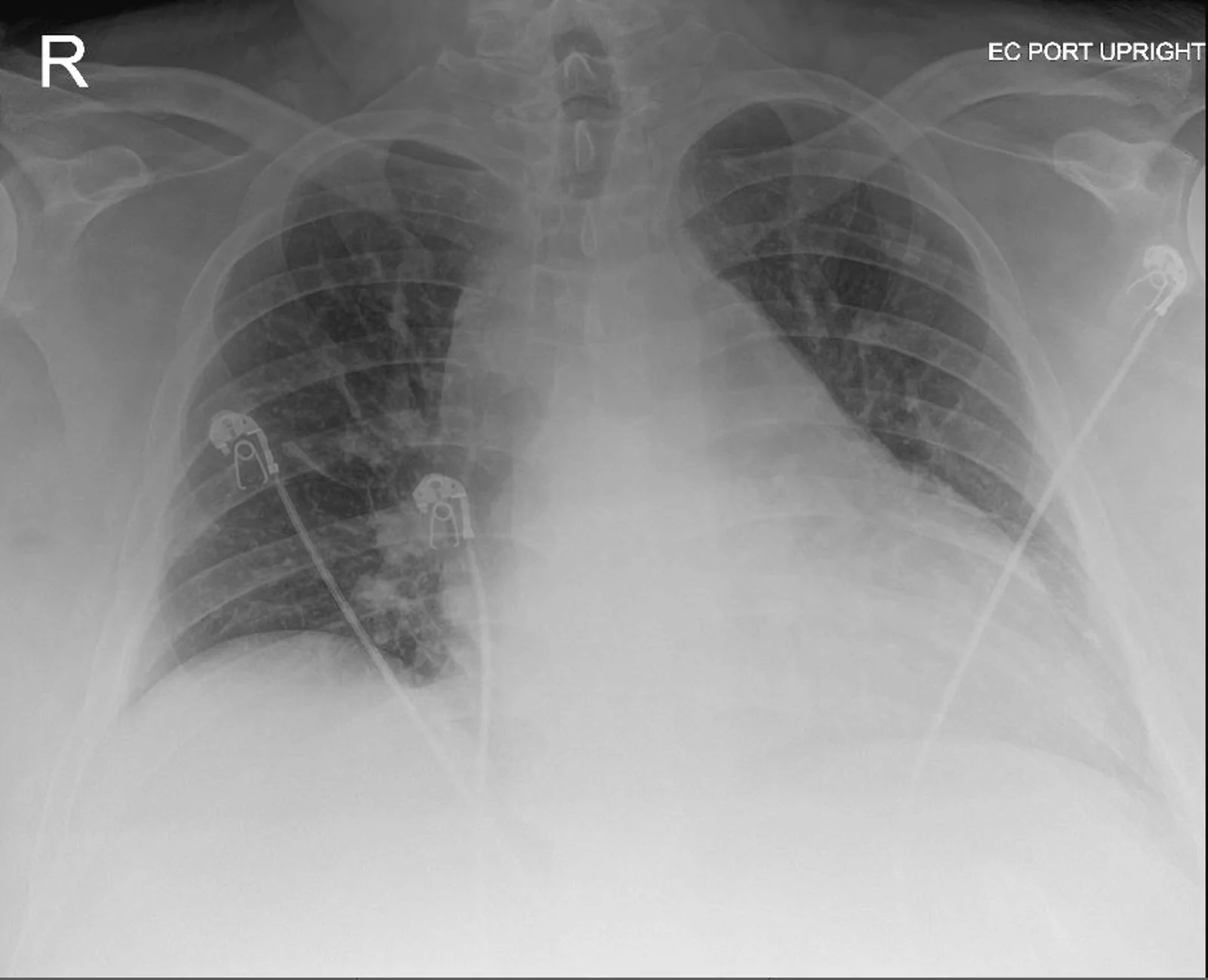
Admission chest radiograph. Portable upright anteroposterior (AP) chest radiograph showed an enlarged cardiac silhouette and mild pulmonary vascular congestion. AP projection can magnify the cardiac silhouette, limiting assessment of true cardiac enlargement.

Diagnostic shift and point-of-care ultrasound

After an additional 250-mL crystalloid bolus, he received midodrine 10 mg orally, with a transient improvement in blood pressure. His mean arterial pressure then fell again to 60-65 mmHg. Focused cardiac point-of-care ultrasound at the bedside showed a large circumferential pericardial effusion (Figure 3). Computed tomography of the chest, abdomen, and pelvis obtained during the same evaluation also showed a large pericardial effusion, which radiology described as possibly hemorrhagic, without an acute intra-abdominal explanation (Figure 4). As his blood pressure continued to fall and dizziness developed, the working diagnosis shifted toward obstructive shock, and interventional cardiology was contacted urgently.

**Figure 3:**
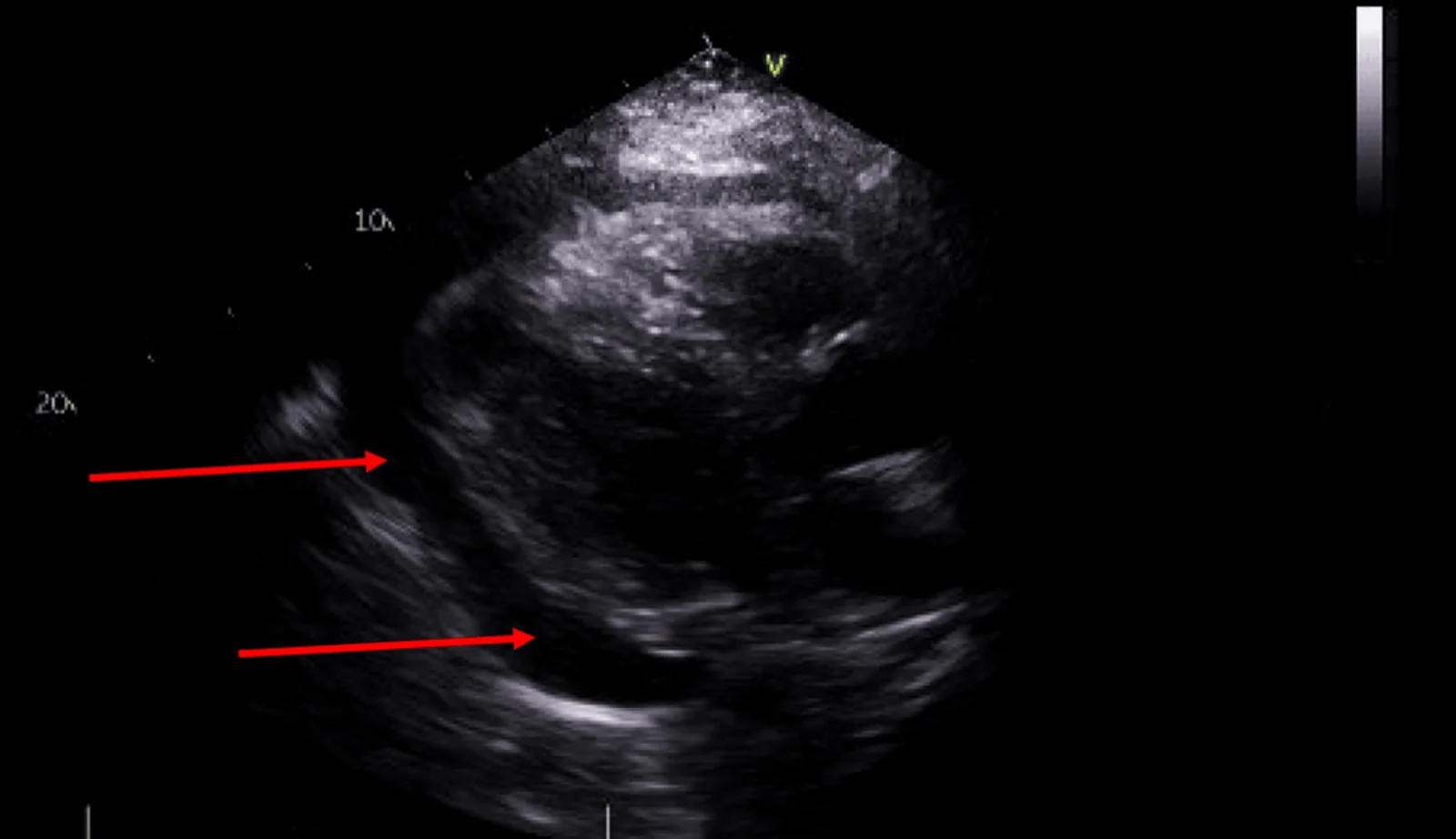
Bedside POCUS demonstrating a large pericardial effusion. Parasternal long-axis bedside cardiac ultrasound showing a large pericardial effusion. The red arrows indicate anechoic pericardial fluid surrounding the heart. POCUS: point-of-care ultrasound

**Figure 4:**
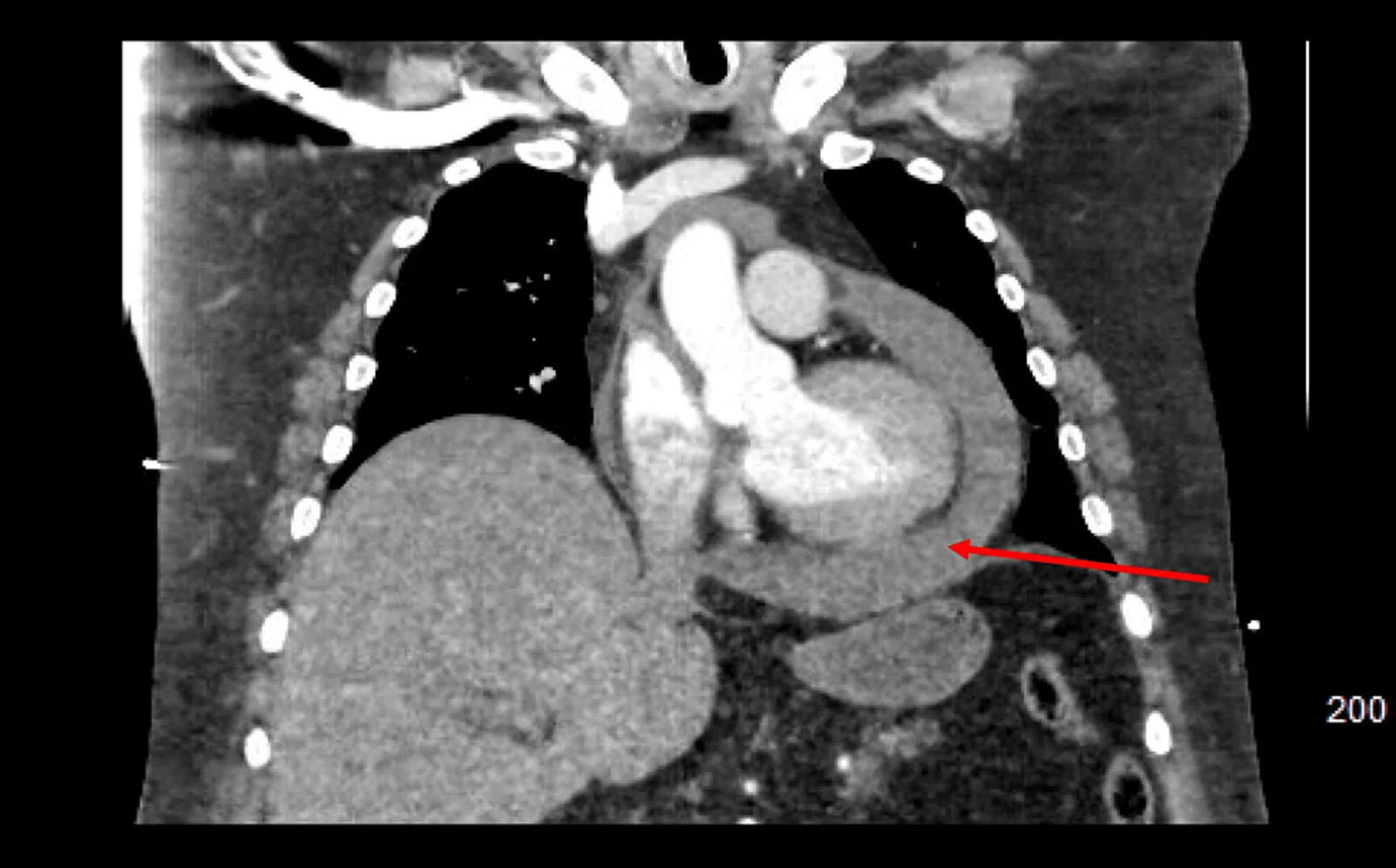
Computed tomography demonstrating a large pericardial effusion. Coronal contrast-enhanced computed tomography of the chest demonstrates a large pericardial effusion (arrow). The radiology interpretation described the effusion as possibly hemorrhagic.

Norepinephrine was started, and the catheterization laboratory was activated for emergent pericardiocentesis. Limited echocardiography showed that the effusion was predominantly posterior. Percutaneous drainage was attempted through subxiphoid and apical approaches but was unsuccessful because there was no adequate anterior fluid pocket. Further attempts were limited by the patient's body habitus and bleeding risk in the setting of recent apixaban exposure and an elevated international normalized ratio.

Therapeutic intervention and hospital course

Shortly after transfer to the intensive care unit, the patient developed pulseless electrical activity arrest. Return of spontaneous circulation was achieved after approximately 10-11 min of cardiopulmonary resuscitation, and he was intubated. Repeat echocardiography showed right ventricular compression, right atrial inversion, and respiratory variation, findings that supported tamponade physiology (Figure 5).

**Figure 5:**
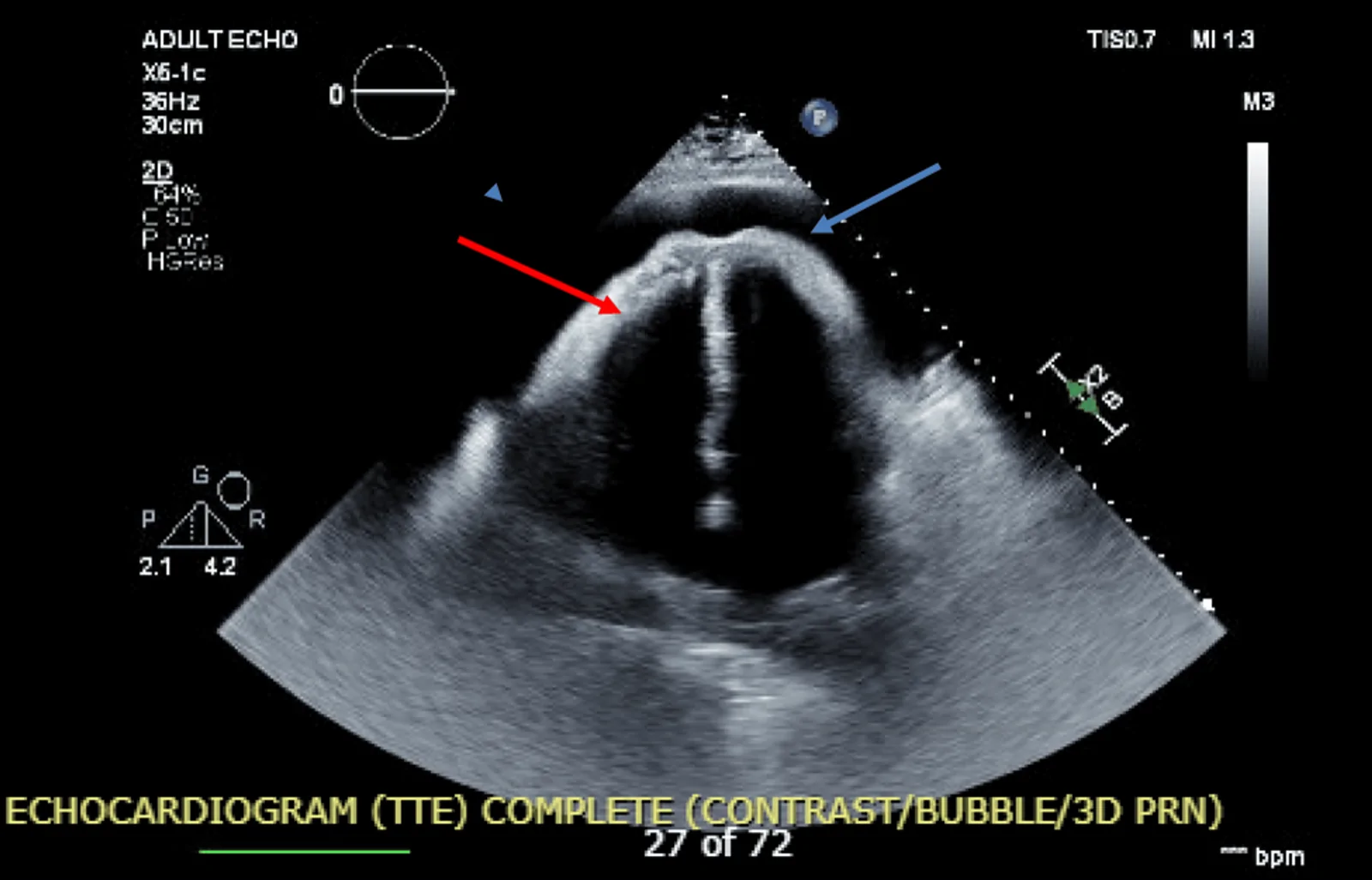
Transthoracic echocardiography demonstrating a large pericardial effusion. Apical transthoracic echocardiographic view demonstrating a large pericardial effusion (blue arrow) and an increased right ventricular-to-left ventricular size ratio (red arrow). Repeat echocardiography demonstrated right ventricular compression, right atrial inversion, and respiratory variation, supporting tamponade physiology. TTE: transthoracic echocardiography; PRN: as needed

Table 1 summarizes the classic ultrasound findings of tamponade and indicates which were documented in this patient [13]. Cardiothoracic surgery performed an emergent subxiphoid pericardial window with drain placement. Approximately 450 mL of bloody pericardial fluid was removed, followed by hemodynamic improvement.

**Table 1:** Classic point-of-care ultrasound and echocardiographic findings that support tamponade physiology. This table was created by the authors of this study. Cardiac tamponade remains a clinical diagnosis, and no single ultrasound finding is diagnostic in isolation [13].

Findings	Typical ultrasound finding	Documented in this case
Pericardial effusion	Anechoic or hypoechoic fluid surrounding the heart	Large circumferential effusion; predominantly posterior
Right atrial systolic collapse or inversion	Inward motion of the right atrial free wall during systole	Right atrial inversion present
Right ventricular diastolic collapse or compression	Inward motion of the right ventricular free wall during early diastole	Right ventricular compression present
Respiratory variation in transvalvular inflow	Exaggerated respiratory variation in mitral and tricuspid Doppler inflow	Respiratory variation reported; valve-specific measurements unavailable
Plethoric inferior vena cava	Dilated inferior vena cava with reduced inspiratory collapse	Not documented

The patient required mechanical ventilation, vasopressor support, and continued hemodialysis during his intensive care unit course. Pericardial fluid was described by the laboratory as red and bloody, with 71,100 red blood cells/µL and 6,800 nucleated cells/µL. Cytology showed abundant blood without malignant cells, and fluid cultures were negative. Pericardial fluid hematocrit and hemoglobin were not available in the records reviewed. Whether the fluid was clotted was not documented. Blood cultures from two peripheral draws showed no growth at five days, and antibiotics were discontinued after infection was excluded. Follow-up echocardiography showed resolution of the pericardial effusion, and the drain was removed on hospital day three.

Cardiology recommended holding apixaban during acute management because of concern for hemorrhagic effusion. No administration of andexanet alfa was documented. Because the immediate threat was obstructive shock, the patient proceeded to mechanical drainage. The discharge summary instructed continuation of apixaban 5 mg twice daily, noting that hemoglobin was at baseline and recommending primary care monitoring. The available records did not specify the inpatient restart date, the rationale for resuming this dose, or whether cardiology or nephrology participated in that decision. He was discharged to a skilled rehabilitation facility after an 11-day hospitalization.

## Discussion

Obstructive shock should be considered during the initial clinical evaluation of undifferentiated hypotension. In an anticoagulated patient with unexplained shock, hemorrhagic pericardial effusion with tamponade should be considered. The diagnosis was not obvious at presentation. Hypotension began during dialysis, and the main symptom was abdominal pain. Sepsis was initially considered, although leukocytosis and elevated lactate were nonspecific and did not distinguish the cause of shock. The ED examination documented no jugular venous distention, whereas cardiology subsequently recorded a jugular venous pressure of 12-14 cm. Bedside POCUS identified a large pericardial effusion, shifting the working diagnosis toward obstructive shock. CT subsequently described the effusion as possibly hemorrhagic, and formal echocardiography followed CT. Focused cardiac ultrasound should be incorporated into the initial evaluation of undifferentiated shock [14,15].

Hemorrhagic tamponade associated with direct oral anticoagulants is rare. Published reports often describe patients with renal dysfunction, coagulation abnormalities, advanced age, or interacting medications [10,11]. This patient had end-stage kidney disease and anemia, and uremia itself can impair platelet function. Hemodialysis often uses unfractionated or low-molecular-weight heparin to prevent clotting in the extracorporeal circuit, while repeated access cannulation adds another opportunity for bleeding [16]. However, the available dialysis-center transfer documentation was incomplete, and it could not be determined whether heparin had been administered during the index dialysis session. Therefore, its contribution to bleeding cannot be established. His medication list contained no aspirin, other antiplatelet agent, or nonsteroidal anti-inflammatory drug. 

A single case cannot establish that full-dose apixaban caused the hemopericardium. The dose is still clinically relevant because the patient was taking 5 mg twice daily and had taken the morning dose. In a steady-state pharmacokinetic study of hemodialysis patients, Mavrakanas et al. found that only about 4% of apixaban was removed during dialysis after a 2.5-mg dose and that 5 mg twice daily produced exposure above the range seen in patients with preserved renal function [6]. Studies in peritoneal dialysis have also found little dialytic clearance, marked variation between patients, and high drug exposure despite reduced dosing [7,8]. These data do not establish causation in this patient, but they support caution in dose selection.

Outcome studies have not resolved the issue. In observational Medicare data, apixaban was associated with less major bleeding than warfarin, and standard-dose apixaban was associated with fewer strokes or systemic emboli than reduced-dose apixaban [2]. Because treatment was not randomized, confounding and selection bias remain important. The RENAL-AF trial compared apixaban, predominantly 5 mg twice daily, with warfarin in 154 hemodialysis patients, but it stopped early and was underpowered [3]. The AXADIA-AFNET 8 trial randomized 97 hemodialysis patients to apixaban 2.5 mg twice daily or phenprocoumon and did not provide definitive evidence of superiority or noninferiority [4]. A later pilot trial in Canada and Australia randomized 151 dialysis patients to apixaban 5 mg twice daily, warfarin, or no oral anticoagulation. It showed that a larger trial was feasible but was not designed to identify the best strategy [5]. No single regimen has emerged as clearly preferable for every dialysis patient [9].

Reversal also has limits in a patient with tamponade. Andexanet alfa is a specific reversal agent for life-threatening or uncontrolled bleeding associated with apixaban or rivaroxaban, but its use was not documented in this case. Hemodialysis does not substantially reduce apixaban exposure [17]. Even complete reversal would not relieve the obstructive physiology. Percutaneous drainage was not feasible because most of the fluid was posterior and the available window was limited, so surgical drainage was required. An apixaban-calibrated anti-factor Xa assay can confirm recent drug exposure when available, but the result may not return quickly enough to guide care in an unstable patient and should not delay drainage.

Resuming anticoagulation after hemorrhagic tamponade requires a careful balance between stroke prevention and the risk of recurrent bleeding. Although the discharge prescription was confirmed, the missing restart date, dose rationale, and documentation of specialist involvement limit assessment of the anticoagulation decision. The decision should be individualized and should address stroke risk, recurrent bleeding risk, dose selection, alternatives, and patient preferences, ideally with input from cardiology and nephrology.

## Conclusions

Cardiac tamponade should be considered in any patient with undifferentiated hypotension, including patients whose symptoms begin during hemodialysis. Bedside cardiac ultrasound can identify a pericardial effusion and prompt urgent evaluation for obstructive shock. This case does not prove that apixaban caused the hemopericardium, but it illustrates the uncertainty surrounding full-dose therapy in patients receiving dialysis. After a major bleed, the decision to resume, reduce, or stop anticoagulation should be individualized, and the rationale documented clearly.
